# Procedures for the GMP-Compliant Production and Quality Control of [^18^F]PSMA-1007: A Next Generation Radiofluorinated Tracer for the Detection of Prostate Cancer

**DOI:** 10.3390/ph10040077

**Published:** 2017-09-27

**Authors:** Jens Cardinale, René Martin, Yvonne Remde, Martin Schäfer, Antje Hienzsch, Sandra Hübner, Anna-Maria Zerges, Heike Marx, Ronny Hesse, Klaus Weber, Rene Smits, Alexander Hoepping, Marco Müller, Oliver C. Neels, Klaus Kopka

**Affiliations:** 1 German Cancer Research Center (DKFZ), Division of Radiopharmaceutical Chemistry, Im Neuenheimer Feld 280, 69120 Heidelberg, Germany; y.remde@dkfz.de (Y.R.); martin.schaefer@dkfz.de (M.S.); h.marx@dkfz.de (H.M.); k.weber@dkfz.de (K.W.); o.neels@dkfz.de (O.C.N.); k.kopka@dkfz.de (K.K.); 2 ABX Advanced Biochemical Compounds GmbH, Heinrich-Glaeser-Strasse 10-14, 01454 Radeberg, Germany; rene.martin@abx.de (R.M.); hienzsch@abx.de (A.H.); sandra.huebner@abx.de (S.H.); zerges@abx.de (A.-M.Z.); hesse@abx.de (R.H.); smits@abx.de (R.S.); hoepping@abx.de (A.H.); marco.mueller@abx.de (M.M.); 3 German Cancer Consortium (DKTK), Im Neuenheimer Feld 280, 69120 Heidelberg, Germany

**Keywords:** [^18^F]PSMA-1007, fluorine-18, PSMA, automation, prostate cancer, PET

## Abstract

Radiolabeled tracers targeting the prostate-specific membrane antigen (PSMA) have become important radiopharmaceuticals for the PET-imaging of prostate cancer. In this connection, we recently developed the fluorine-18-labelled PSMA-ligand [^18^F]PSMA-1007 as the next generation radiofluorinated Glu-ureido PSMA inhibitor after [^18^F]DCFPyL and [^18^F]DCFBC. Since radiosynthesis so far has been suffering from rather poor yields, novel procedures for the automated radiosyntheses of [^18^F]PSMA-1007 have been developed. We herein report on both the two-step and the novel one-step procedures, which have been performed on different commonly-used radiosynthesisers. Using the novel one-step procedure, the [^18^F]PSMA-1007 was produced in good radiochemical yields ranging from 25 to 80% and synthesis times of less than 55 min. Furthermore, upscaling to product activities up to 50 GBq per batch was successfully conducted. All batches passed quality control according to European Pharmacopoeia standards. Therefore, we were able to disclose a new, simple and, at the same time, high yielding production pathway for the next generation PSMA radioligand [^18^F]PSMA-1007. Actually, it turned out that the radiosynthesis is as easily realised as the well-known [^18^F]FDG synthesis and, thus, transferable to all currently-available radiosynthesisers. Using the new procedures, the clinical daily routine can be sustainably supported in-house even in larger hospitals by a single production batch.

## 1. Introduction

Over the past few years, PSMA-PET has become a favourable non-invasive imaging method for the diagnosis of prostate cancer by outperforming choline-PET, in particular for recurrent disease with low PSA levels [1,2,3,4,5,6]. In clinical applications, ^68^Ga-labelled PSMA-targeting radioligands [7,8,9] are currently among the most frequently used due to the high number of patients suffering from prostate cancer, which may benefit from a PSMA-PET scan [10,11,12,13,14]. However, the production capacity given by generator-based radionuclide gallium-68 is limited [15]. Consequently, there is a remarkable interest in radiofluorinated PSMA-targeting ligands [15,16,17,18,19].

**Scheme 1:**
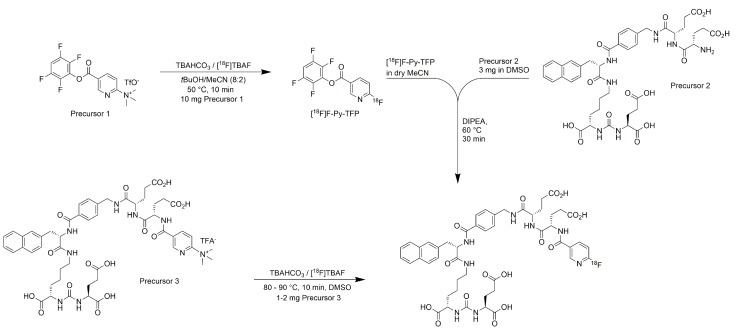
Routes for the radiosynthesis of [^18^F]PSMA-1007 (TBAHCO_3_: tetrabutylammonium hydrogen carbonate; TBAF: tetrabutylammonium fluoride; *t*BuOH: *tert*-butanol; MeCN: acetonitrile; F-Py-TFP: 6-fluoropyridine-3-carboxylic acid 2,3,5,6-tetrafluorophenyl ester; DIPEA: diisopropylethylamine; DMSO: dimethylsulfoxide).

In 2016, we reported the development of [^18^F]PSMA-1007 [15], a highly promising candidate for the detection of prostate cancer by means of PET/CT and PET/MRI [20,21,22,23]. After successful preclinical evaluation [15], our next aim was the development of a straightforward GMP-compliant radiosynthesis for [^18^F]PSMA-1007 using fully-automated radiosynthesisers including suitable procedures for quality control (QC) mandatory for human application [20,21,22,23]. Our first approach, which was also used during the individual first-in-man studies, was an improved version of a two-step procedure already applied during the preclinical development of [^18^F]PSMA-1007 [15] using Precursors 1 and 2 (Scheme 1). Since the radiochemical yields were rather poor, we developed a novel precursor for the synthesis of the tracer by direct nucleophilic substitution [24]. During this development, it turned out that the radiofluorination of [^18^F]PSMA-1007 can be performed selectively, and thus, the unprotected Precursor 3 could be used (Scheme 1).

We herein report on the automation of the original two-step procedure for the production of [^18^F]PSMA-1007 used during the first-in-man studies. Furthermore, we report on the automation of the novel one-step procedure on a number of commonly-used radiosynthesisers (GE TRACERlab FX FN and MX, NEPTIS mosaic-RS and IBA SYNTHERA+). Finally, we suggest procedures for the QC of [^18^F]PSMA-1007 injection solution and acceptance criteria compliant with recent European Pharmacopoeia (Eur. Ph.) standards [25].

## 2. Results

### 2.1. Production of [^18^F]PSMA-1007 via a Two-Step Procedure on a Trasis AllInOne Module

We herein include the results of our first 24 successful production batches for clinical use with activity yields between 300 and 2200 MBq and activity concentrations in the range of 50–220 MBq/mL. The average radiochemical yield (after HPLC, isolated) with respect to the activity trapped on QMA cartridge (Waters, Eschborn, Germany) within the synthesiser was 5.1 ± 2.3% after a total synthesis time of approximately 80 min. 

Despite some minor deviations for ethanol contents of up to 100 mg/mL, none of the productions failed due to acceptance criteria during QC. Regarding residual solvents, the highest value of acetonitrile determined was 156 µg/mL (mean value 17 ± 32 µg/mL), while all other values for acetonitrile were below 60 µg/mL. For dimethylsulfoxide (DMSO), the highest value determined was 164 µg/mL (mean value 32 ± 51 µg/mL), while more than half of the DMSO contents were below the limit of quantitation. Residual *t*BuOH or diisopropylethylamine (DIPEA) was not observed in any batch. Average pH-values were 6.2 ± 0.5. Specific activities were in the range of 10–150 MBq/µg (average value of 58 ± 34 MBq/µg equal to molar activities of 60 ± 36 GBq/µmol) with a radiochemical purity of 97.1 ± 1.3%, showing two minor radioactive contaminations of approximately 3% in sum. All further tests were qualitatively passed or below the limit of quantitation (LOQ). A downstream test for sterility did not reveal any microbial contamination of the formulations. In all cases with ethanol limit exceedance, the responsible physician was informed, who decided to further dilute the formulation with saline before injection. 

**Figure 1:**
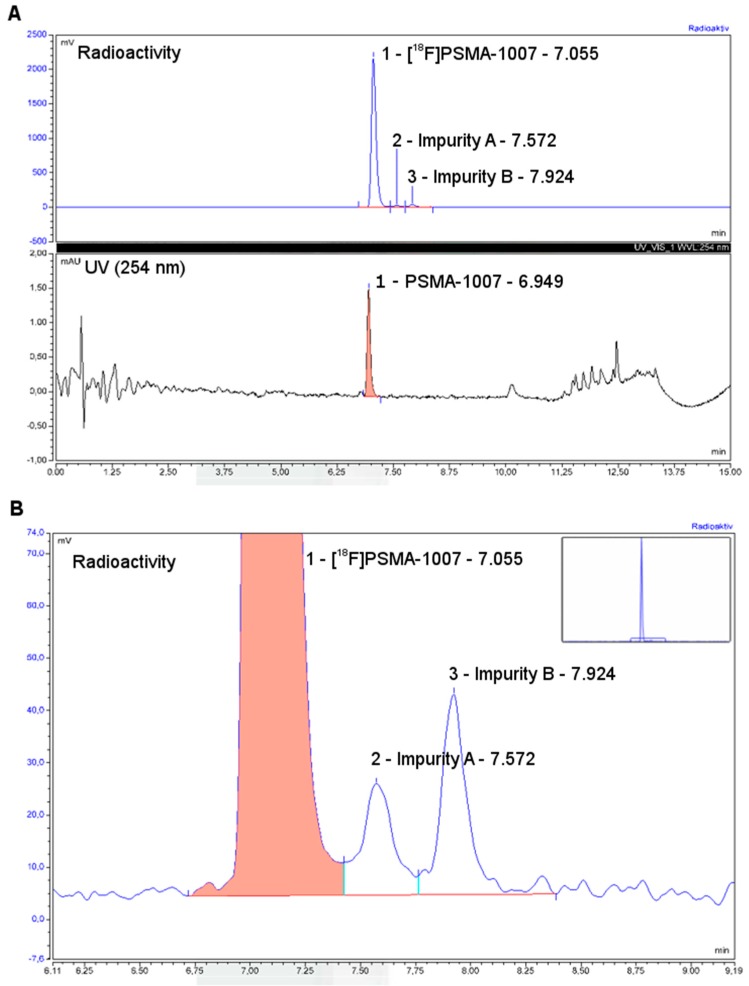
Relevant chromatograms for the release of [^18^F]PSMA-1007 from the two-step production on the AllInOne radiosynthesiser: Full chromatogram of the [^18^F]PSMA-1007 formulation showing co-elution of cold PSMA-1007 and radioactive product (**A**) and zoomed around the radioactive product peak (**B**).

Typical chromatograms from the analytical radio-HPLC of [^18^F]PSMA-1007 after preparation by the two-step process on the AllInOne radiosynthesiser are shown in Figure 1.

### 2.2. Production of [^18^F]PSMA-1007 via Direct Substitution on Tracerlab FX FN Module (SPE Purified)

Included are the results from our first syntheses for the application of the single-step radiofluorination process on synthesisers used for clinical routine production (*n* = 16). After a total synthesis time of 55 min, [^18^F]PSMA-1007 was isolated in radiochemical yields of 24.3–82.4% and activity yields between 5.7 and 15.1 GBq at the end of synthesis as injectable solution, starting from 40 GBq activity. The radiochemical purity was 97.0 ± 1.1% (radio-HPLC). The average amount of non-radioactive PSMA-1007 was 5.9 ± 1.7 µg/mL, equal to a molar activity of 126 ± 42 GBq/µmol (15 mL injection solution). Free fluorine-18 was less than 0.1% (TLC), and residual phase transfer catalyst tetrabutylammonium (TBA) below the limit of 260 µg/mL (semi-quantitative spot test). The amounts of residual solvents were below the pre-defined limits (acetone 134.1 ± 150.9 µg/mL; acetonitrile 5.3 ± 3.7 µg/mL; DMSO 5.5 ± 14.3 µg/mL). The pH of the final formulation was 5.1 ± 0.4. Sterility and bacterial endotoxin testing and gamma spectroscopy were performed against the defined specifications. 

### 2.3. Production of [^18^F]PSMA-1007 via Direct Substitution on GE TRACERlab MX and NEPTIS Mosaic-RS (SPE Purified)

For upscaling of the synthesis, several high-activity runs (*n* = 10) were performed with initial activities of up to 80 GBq, delivering [^18^F]PSMA-1007 in activity yields of up to 30 GBq. The radiochemical yields were in the range of 43.3–52.8% on the TRACERlab MX and 41.3–44.9% on the mosaic-RS synthesiser, respectively, after an overall synthesis time of 45 min. Radiochemical purity determined by HPLC was ≥95% after 8 h. Free fluorine-18 was less than 1% (TLC) after 8 h. All remaining chemical impurities were ≤0.1 mg/V_max_, and the ethanol content was in the range of 6.8–7.1% *v*/*v*. The pH of the final solution was between 5.5 and 6.0. 

### 2.4. Production of [^18^F]PSMA-1007 via Direct Substitution on IBA SYNTHERA+ (SPE Purified)

**Figure 2:**
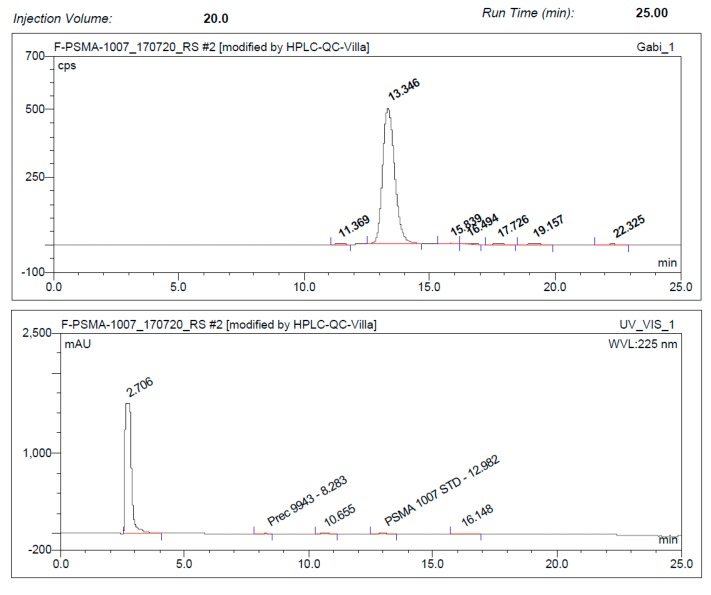

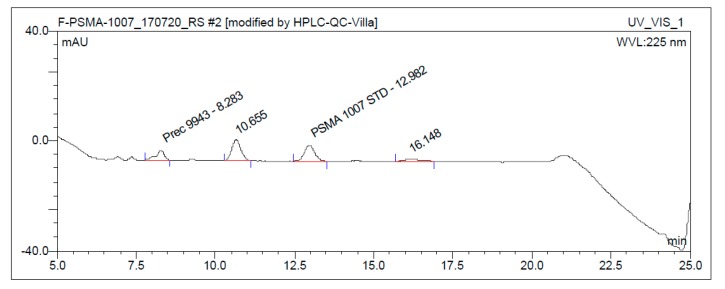
Typical chromatographic radio- and ultraviolet (UV)-traces of [^18^F]PSMA-1007 injection solution (ABX advanced chemical compounds) using the one-step method (Prec. = Precursor).

**Figure 3:**
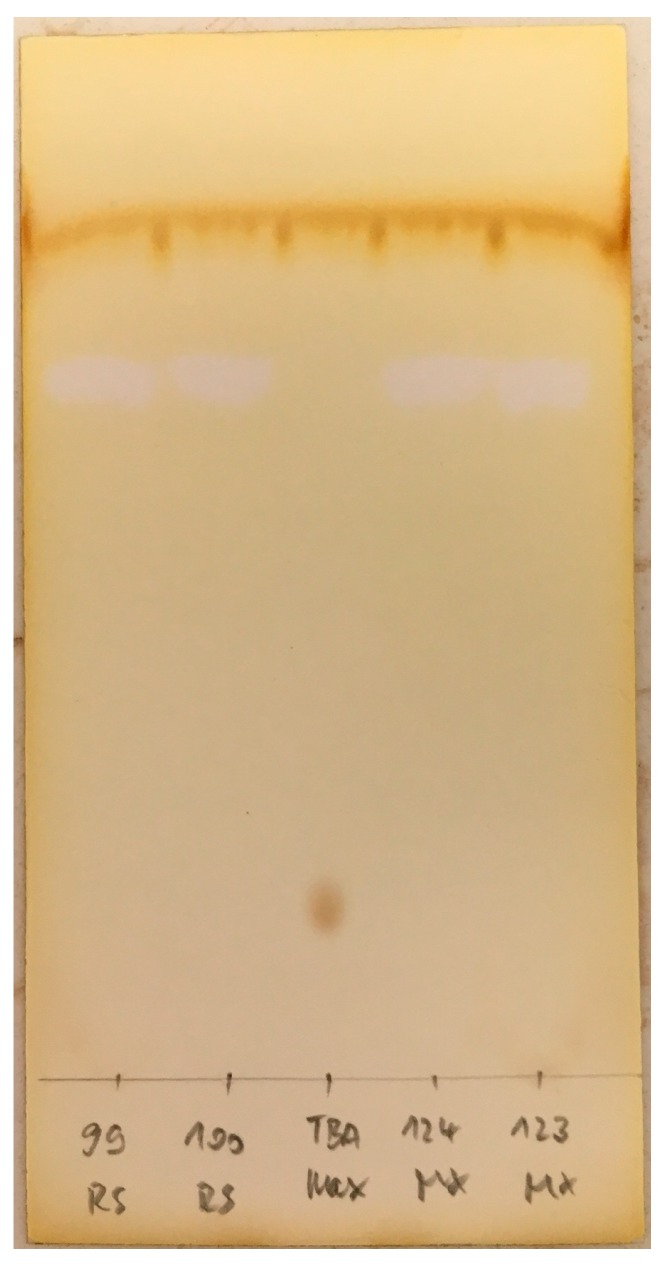
Iodine-stained TLC from the tetrabutylammonium (TBA) test showing the reference solution 0.1 mg/mL in the middle and respective final solutions from TRACERlab MX (**left**) and mosaic-RS (**right**).

On the IBA SYNTHERA+, [^18^F]PSMA-1007 was produced in radiochemical yields between 59.5% and 72.8% after an overall synthesis time of 35 min using activities of up to 89 GBq. Activity yields were up to 49 GBq. Radiochemical purity was ≥95% after 8 h (radio-HPLC analysis, Figure 2). Free fluorine-18 was less than 1% (TLC) after 8 h (Figure 3). Chemical impurities were ≤0.1 mg/V_max_, and ethanol content was in the range of 6.8–7.1% *v*/*v*. The content of TBA was below LOQ in all of the final solutions.

## 3. Discussion

### 3.1. Production of [^18^F]PSMA-1007 via a Two-Step Procedure on Trasis AllInOne Radiosynthesiser

It was already known from our preclinical experiments that [^18^F] 6-fluoropyridine-3-carboxylic acid (F-Py-TFP) exhibits a bad reactivity towards Precursor 2 (conjugation yield of [^18^F]F-Py-TFP to the amino group of glutamic acid was actually always low compared to other amino groups) [26]. We assume that the amino-group in Precursor 2 is deactivated, most probably by lactamisation. An alternative precursor with protected carboxylic acid(s), at least in the terminal position, should lead to an improvement of the yield, however also necessitating an additional step for deprotection. Therefore, our preliminary experiments focused on improving the reaction conditions. The initial radiolabeling reaction delivering [^18^F]F-Py-TFP proved to be optimal under the conditions reported by Olberg et al. [27]. However, we slightly increased the reaction temperature to 50 °C and have chosen a reaction time of 10 min. It turned out that the yield of the conjugation to Precursor 2 could be increased under dry conditions using DIPEA as the base (pyridine, triethylamine and Kryptofix 2.2.2/K_2_CO_3_ were also tested, but led to decreased yields), which was then used for full automation. Since the yield obtained by this procedure was sufficient for the first-in-man studies, no alternative precursor was considered at this stage.

**Figure 4:**
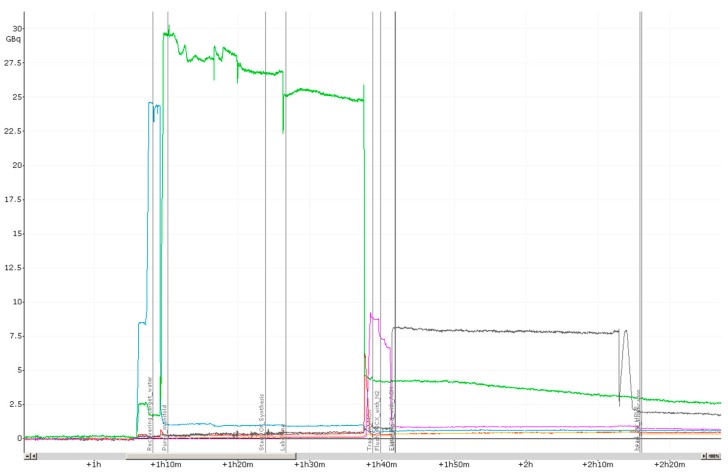
Activity trails during a typical production of [^18^F]PSMA-1007 by the two-step procedure on Trasis AllInOne radiosynthesiser.

The activity trails recorded by the five sensors inside the synthesiser are shown in Figure 4. The position of the detector probes, as well as their colour code are indicated in Figure 6 (see below in Materials and Methods) by the radiation symbols and the respective coloured fields below.

The blue line indicates the activity trapped on the QMA-cartridge. As expected, the activity is efficiently eluted to the reaction vessel using TBAHCO_3_-solution (green trail). The green trail (activity in Reaction Vessel 1) shows some noisy behaviour during the drying process, which is caused by the changes of the activity per volume in Vessel 1 occurring throughout the process (geometric factors). About 20% of the radioactivity is left in Reaction Vessel 1 after labelling and extraction of the mixture, which is a behaviour for no carrier added [^18^F]F^−^ typically observable in glass reaction vessels [28]. Roughly 30% of the activity from Reaction Vessel 1 is then trapped on the MCX cartridge (purple line). Actually, we expected a somewhat higher value of adsorption of approximately 45% at this stage. This deviation may be caused by sputtering of the reaction mixture during the drying process, resulting in uncomplete contact of the reaction solvent with the activated [^18^F]F^−^-complex in Reaction Vessel 1. Further, a small loss of activity is caused by the first elution of the cartridge with 500 µL acetonitrile. However, it was known from preliminary experiments that this fraction elutes with unidentified small particles presumably originated from the cartridge material. Considering the following cartridge drying, final HPLC purification and sterile filtration, there was no risk for contamination of the final injection solution at all; however, with respect to the risk of clogging the cassette at one of those barriers, we decided to discard this fraction anyway. Finally, [^18^F]F-Py-TFP is eluted from the cartridge into Reaction Vessel 2 (grey line) for the second step of the labelling procedure (conjugation to Precursor 2). Obviously, there is no unexpected behaviour except for the “peak” at the end of the grey activity trail, which is also caused by geometric factors.

**Figure 5:**
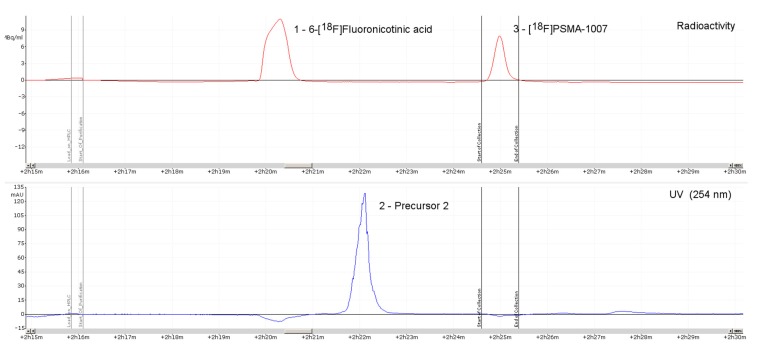
Radio-chromatogram of the [^18^F]PSMA-1007 separation on integrated HPLC of the Trasis AllInOne radiosynthesiser.

Before HPLC purification, the basic reaction mixture in Reaction Vessel 2 containing the acidic [^18^F]PSMA-1007 had to be acidified and diluted. Therefore, addition of 6 mL water containing 10 µL TFA proved to be sufficient. All major impurities including 6-[^18^F]Fluoronicotinic acid (formed by hydrolysis of [^18^F]F-Py-TFP), non-reacted Precursor 2 and [^18^F]F-Py-TFP were effectively separated by the final semi-preparative HPLC (Figure 5). However, it turned out that two minor radiochemical impurities are formed during the reaction (altogether approximately 3%), which could not be separated by HPLC. We believe that addressing this problem would at least require a solvent change to an acetonitrile/acidified water mixture. Furthermore, measures to decrease injection volume or even a solvent change before injection aiming towards higher resolution of the semi-preparative HPLC could be necessary, which would add substantially to the complexity of the process. Therefore and because of the low levels of impurities, we decided to accept those side products. In none of the batches produced by the described method were significant chemical impurities observed.

In summary, we successfully and reliably produced 24 batches of [^18^F]PSMA-1007 for first-in-man PET/CT studies. Although yields for the reaction presented here are low, the general feasibility of a two-step radiofluorination with the prosthetic group [^18^F]F-Py-TFP using an AllInOne module has been demonstrated. Impurities arising from [^18^F]F^−^ activation during the initial labelling step are effectively separated by the cartridge extraction process before subsequent coupling of the prosthetic group. Anyhow, for precursors showing a better reactivity towards [^18^F]F-Py-TFP, we estimate that on a daily basis, a multi-dose batch production of the respective radioligands is feasible. Furthermore, the procedure proved to be excellent for the setup of respective new libraries of radiotracer variants bearing the 6-[^18^F]Fluoropyridine-3-carboxy moiety as a radiolabel-bearing subunit, as well as the preliminary (“bridging”) synthesis procedure during clinical translation.

### 3.2. Production of [^18^F]PSMA-1007 by Direct One-Step Synthesis on the GE Tracerlab FX FN Module, GE TRACERlab MX, NEPTIS Mosaic-RS and IBA SYNTHERA+

Although the used synthesisers are quite different, procedures for the production of [^18^F]PSMA-1007 injection solution by direct radiofluorination are still quite comparable on the selected systems and therefore discussed together.

The reaction proved to be reproducible, delivering the product in good radiochemical yields between 25% and 80% after cartridge separation with slightly higher yields on the IBA SYNTHERA+. The higher yield on the SYNTHERA+ is most probably due to shorter fluidic pathways causing fewer losses in tubings and manifolds. On all modules, the product was obtained in excellent synthesis times well below 55 min. Upscaling of the synthesis using start activities of approximately 90 GBq resulted in activity yields of up to 49 GBq and was finally accomplished on IBA SYNTHERA+. During upscaling, no affection of the radiochemical yield was observed. However, it should be noted that the addition of sodium ascorbate as a stabiliser to avoid radiolysis in the final formulation is necessary when product activities of more than 20 GBq are produced (threshold activity concentration of approximately 1 GBq/mL). Using sodium ascorbate addition stability was proven over a time period of 8 h, which is a typical shelf-life for fluorine-18 radiopharmaceuticals (in prior experiments, 100 mg were used).

Importantly, the cartridge separation is the crucial step for the quality of the final product. During this separation process, multiple subtle washing steps have to be applied. After fixation of the product and impurities from the crude reaction mixture, the more hydrophilic side products are removed by washing with 5% EtOH solution in a first step. Subsequently, the product is fractionally eluted with 30% EtOH (25% EtOH in the case of IBA SYNTHERA+). The first 30% EtOH fraction (3 mL) is still contaminated with significant amounts of impurities and therefore has to be discarded. Furthermore, the volume of the second fraction (5 mL) was adjusted (limited) for avoiding the introduction of more lipophilic impurities. Using this approach, impurities were only present in trace amounts well below the limits according to recent Eur. Ph. monographs (important note: acceptance criteria were chosen based on typical monographs for fluorine-18 radiopharmaceuticals (e.g., [^18^F]FET Monograph No. 07/2015:2466 Eur. Ph.) and the monograph on radiopharmaceutical preparations (Monograph No. 07/2016:0125)). Therefore, we recommend using these specifications when applying the radiosynthesis in a PET radiopharmacy. Furthermore, one should be aware that the product quality can be further improved by careful adjustment of the volumes for washing and eluting the cartridges. Application of typical quality control procedures for the release of radiopharmaceuticals (see below) revealed that all produced batches could have been released for clinical use without restriction of any kind.

Principally, the product can also be purified by suitable semipreparative HPLC procedures. However, the additional time including separation using the mobile phase and reformulation of the product can be estimated with approximately 45 min, which equals 25% product loss owing to decay only.

In summary, we developed a precursor and a unique synthetic procedure for the highly economic production of [^18^F]PSMA-1007 injection solution, [^18^F]PSMA-1007 being the next generation ^18^F-tracer for the diagnosis and noninvasive staging of PSMA-positive prostate cancer. The produced batches meet all acceptance criteria according to recent Ph. Eur. Upscaling was successfully conducted to batch sizes of approximately 50 GBq with proven stability over 8 h. Thus, the clinical routine even in larger hospitals can be sustainably supplied on a daily basis by single batches of [^18^F]PSMA-1007 obtained by the novel one-step radiofluorination procedure transferable to commercially available radiosynthesisers.

### 3.3. Quality Control

#### 3.3.1. Acceptance Criteria

Acceptance criteria were chosen in compliance with the general texts and monographs of the current European Pharmacopoeia and are summarised in Table 1. Most of the QC methods are standard procedures for skilled personnel and, thus, do not need to be discussed here. However, special emphasis should be given to the chemical purity of the product. Limits were also chosen comparable to existing monographs for fluorine-18-labelled radiopharmaceuticals and are 0.1 mg/V_max_ for PSMA-1007 (^19^F-carrier), not more than 0.1 mg/V_max_ for a single unknown impurity assuming the same extinction coefficient like PSMA-1007, the sum of all unknown impurities including PSMA-1007 not more than 0.5 mg/V_max_ and a disregard limit of 0.03 mg/V_max_ for any unknown impurity detected in analytical HPLC. We recommend a minimal radiochemical purity of 95% of the total activity based on TLC for free fluoride-18 and HPLC for any other radiochemical impurity for the release of [^18^F]PSMA-1007. 

#### 3.3.2. Discussion of Quality Control

With respect to the one-step procedure with cartridge separation, a tolerable amount of side-products will occur in the final formulation. Using the limits as recommended, all produced batches from the direct radiolabeling method fulfill the release criteria.

In all productions (one- and two-step), the amount of carrier PSMA-1007 was in the range of 1–10 µg/mL. For research purposes, we calibrated in the range of 0.5–20 µg/mL; however, considering the chosen V_max_ of 10 mL/patient, a calibration curve covering a 3–10-µg/mL range is sufficient with respect to the limit of non-radioactive PSMA-1007 content and the disregard limit. The standard wavelength of 254 nm using the aromatic absorption and/or 220 nm should be applied. Since measurements using the standard wavelengths proved to be sufficient, no additional UV-spectrum for the determination of absorption maxima was recorded.

A test for osmolality using freeze point reduction could not be applied due to the ethanol content of the final formulation. Anyhow, intravenous injectability of the final injection solution is guaranteed by the application of 0.9% saline as the main component (70%) in the chromatographic separation and final dilution (1:3) with isotonic PBS, resulting in a final ethanol concentration of 7.5% *v*/*v*. 

In the case of the two-step procedure, additional tests for *t*BuOH and DIPEA by GC were applied. In all tests, contents were below LOQ. 

It was shown that TBA can be precisely quantified using the TLC spot test presented above (Figure 3). This represents a quick and low-cost method for analysis of TBA as compared to the liquid chromatography currently described in Ph. Eur. monographs. The TLC test is currently under evaluation and hopefully will be published soon in the Ph. Eur. as an alternative to the HPLC test method.

## 4. Materials and Methods 

### 4.1. Production of [^18^F]PSMA-1007 via a Two-Step Procedure on a Trasis AllInOne Module

#### 4.1.1. Setup of the Radiosynthesiser

**Figure 6:**
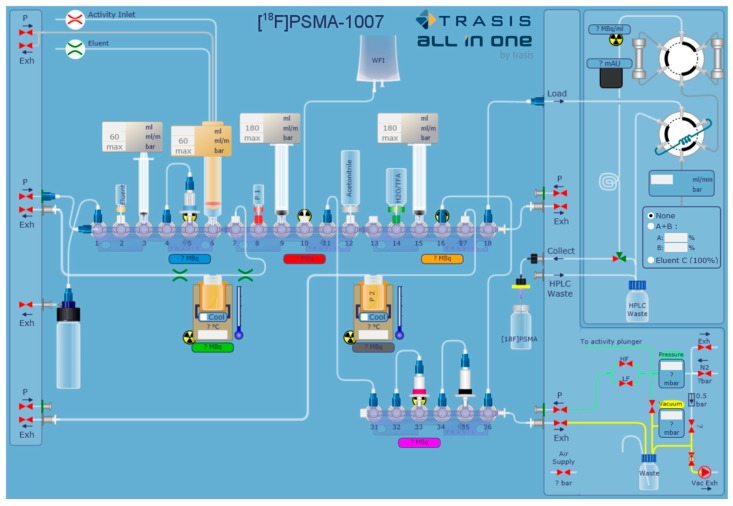
General setup of the synthesis cassette for two-step production of [^18^F]PSMA-1007 with HPLC purification on AllInOne (Exh: Exhaust to vacuum pump).

The radiosyntheses were conducted on an AllInOne PET tracer radiosynthesiser (Trasis, Liege, Belgium) with 36 manifold actuators and an integrated HPLC (Trasis) equipped with a Chromolith Performance RP18_e_ column (100 × 10 mm). The general setup of the cassette is depicted in Figure 6 showing connections of tubings, syringes and other disposables. The cassettes were assembled in-house from standard disposable materials supplied by Trasis. Briefly, 3 mL (Position 3) and 10 mL (Positions 9 and 15) Luer lock syringes (Becton Dickinson, Heidelberg, Germany) were used on the syringe actuators. For cartridge separations, a Sep-Pak^®^ Light Accell^TM^ Plus QMA Cartridge (Waters, Eschborn, Germany) (preconditioned with 5 mL 1 M K_2_CO_3_ followed by 10 mL water) and an Oasis^®^ MCX Plus Extraction Cartridge (Waters) (preconditioned with 5 mL 1 M HCl followed by 5 mL acetonitrile and 10 mL water) were installed on the cassette in Positions 5 and 33, respectively. In Position 35, a Sep-Pak^®^ Dry Cartridge (unconditioned Na_2_SO_4_) was installed.

#### 4.1.2. Reagents

The synthesis of Precursor 1 was accomplished as described by Olberg et al. [27]. The syntheses of Precursor 2 and reference standard PSMA-1007 were accomplished as previously described [15]. Tetrabutylammonium hydrogen carbonate (TBAHCO_3_)-solution, as well as phosphate-buffered saline (PBS) of GMP grade were provided by ABX advanced biochemical compounds (Radeberg, Germany). Potassium carbonate and 1 M hydrochloric acid (both pro analysis), as well as acetonitrile (for DNA synthesis) were purchased from VWR international (Darmstadt, Germany). Dry DMSO, dry *t*BuOH and diisopropylethylamine (DIPEA) were acquired from Sigma-Aldrich (Munich, Germany), trifluoroacetic acid (TFA) (for peptide synthesis) from Carl-Roth GmbH (Karlsruhe, Germany) and water for injection (pharmaceutical grade) from Fresenius Kabi (Bad Homburg vor der Höhe, Germany). The HPLC-solvent was mixed from ethanol (VWR international), 0.9% NaCl (BBraun, Melsungen, Germany) and acetic acid (Sigma-Aldrich), all pharmaceutical grade. 

Fluorine-18 was produced by irradiation of [^18^O]H_2_O (Rotem Industries Ltd., Arava, Israel) with 16.5-MeV proton beams by ^18^O(p,n)^18^F nuclear reaction. Irradiations were performed with the Scanditronix MC32NI cyclotron at the Division of Radiopharmaceutical Chemistry, German Cancer Research Center (DKFZ, Heidelberg, Germany).

#### 4.1.3. Process Description

The target water containing [^18^F]F^−^ was collected on the radiosynthesiser via the plunger in a 20-mL syringe body without a punch attached to Valve 6. From there, the irradiated [^18^O]H_2_O was passed through the QMA-cartridge in Position 5 into a vessel for recovery of the enriched water in vacuo while the [^18^F]F^−^ was trapped on the QMA-cartridge. Subsequently, the [^18^F]F^−^ was eluted with 600 µL 0.075 M aqueous TBAHCO_3_-solution (Position 2) into Reaction Vessel 1 using the syringe in Position 3 and dried by distillation applying −800 mbar vacuum (exhaust side) and +600 mbar N_2_ pressure (pressures in this synthesiser are given relative to normal pressure) at high flow applying 100 °C to Reaction Vessel 1. After the first distillation step, the manifolds from Valves 3–18 were flushed with dry acetonitrile from Position 12 for removal of water traces from the system using Syringe 3 and subsequently flushed with nitrogen. Then, two azeotropic distillations were performed by the addition of 1.5 mL acetonitrile to Reaction Vessel 1 via Syringe 3 (120 °C for the first distillation and 100 °C for the second; −800 mbar vacuum and 800 mbar N_2_ pressure (high flow) for both steps). Fluoride activation was completed by applying the maximum achievable vacuum (−1000 mbar) to Reaction Vessel 1 for one minute. Then, Precursor 1 (Position 8), dissolved in 1.5 mL *t*BuOH/MeCN 8:2 (*v*/*v*) was added (Syringe 9) to Reaction Vessel 1, and the reaction was allowed to proceed for 10 min at 50 °C.

The reaction was quenched by the addition of 5 mL water, and the resulting mixture passed through the MCX cartridge in Position 33 using Syringe 9, washed with 10 mL water and dried by a stream of nitrogen (500 mbar N_2_ high flow vs. −500 mbar vacuum). Subsequently, the cartridge was firstly eluted with 500 µL dry acetonitrile into the waste for removal of impurities. Then, [^18^F]F-Py-TFP (c.f. Scheme 1) was eluted with 1.8 mL of dry acetonitrile followed by flushing with 500 mbar N_2_ (high flow) via the drying cartridge into Reaction Vessel 2 for subsequent coupling with 3 mg Precursor 2 (cf. Scheme 1) in 100 µL dry DMSO and 10 µL DIPEA in 100 µL dry acetonitrile (precursor solution containing DIPEA were filled into Reaction Vessel 2 during cassette setup). After proceeding for 30 min at 60 °C, the reaction was quenched by the addition of 6 mL water containing 10 µL TFA. The mixture was then transferred to the injection loop of the integrated HPLC system and subsequently purified by semipreparative radio-HPLC (Merck Chromolith RP18e 100 × 10 mm) using a mixture of ethanol, water and acetic acid (300:700:1 *v*/*v*/*v*) as the mobile phase at a flow rate of 4 mL/min. The purified [^18^F]PSMA-1007 was directly transferred to a sterile product vial via a Cathivex-GV 0.22-µm sterile filter. Finally, the product was diluted with PBS in a laminar flow cabinet for pH adjustment and reduction of ethanol concentration to 7–8%.

### 4.2. Production of [^18^F]PSMA-1007 via Direct Substitution on Modified Tracerlab FX FN Radiosynthesiser (Former Nuclear Interface FDG Radiosynthesiser) (SPE Purified)

#### 4.2.1. Setup of the Radiosynthesiser

**Figure 7:**
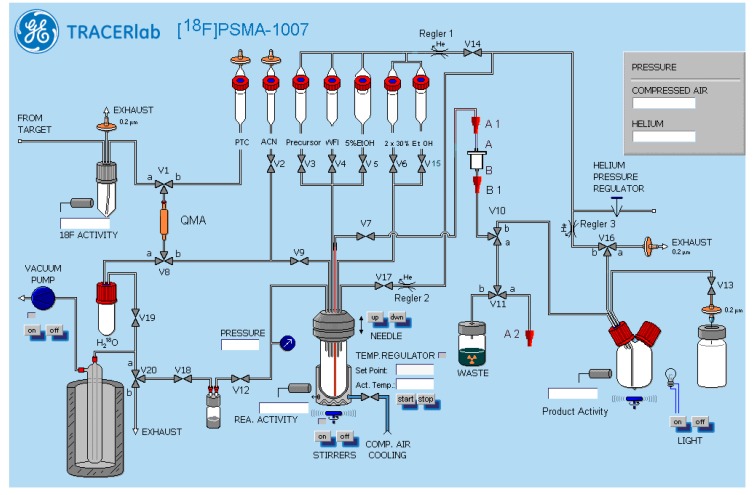
General setup of the radiosynthesiser for the one-step production of [^18^F]PSMA-1007 with SPE cartridge purification on the Nuclear Interface Tracerlab FX FN system.

A Nuclear Interface [^18^F]FDG radiosynthesiser was modified to the needs of a subsequent purification by a setup of two SPE cartridges and formulation into an injectable solution for patient application. GE Tracerlab software was used to write the synthesis sequence and to control the radiosynthesiser. The general setup of the synthesiser is depicted in Figure 7. For cartridge separations, a pre-conditioned Sep-Pak^®^ Light Waters Accell^TM^ Plus QMA cartridge (acquired pre-conditioned from ABX advanced biochemical compounds, Radeberg, Germany), a Chromafix PS-H^+^ (L) cartridge and a Chromafix C18_ec_ (M) (both Macherey-Nagel, pre-conditioned with 3 mL ethanol and 25 mL 5% EtOH) were installed on the radiosynthesiser (QMA between V1 and V8, from reaction vial Chromafix: PS-H^+^ first, C18_ec_ second, between V7 and V10). Connectors A1, A2 and B1 are for reassembly of the system in alternative production configurations with A2 being unused in the configuration discussed here.

#### 4.2.2. Reagents and Radionuclide Production

Precursor 3 and 0.075 M TBAHCO_3_-solution were provided by ABX advanced biochemical compounds (Radeberg, Germany). Acetonitrile (for DNA synthesis) and ethanol were acquired from Merck. Anhydrous DMSO and sodium ascorbate were acquired from Sigma-Aldrich. Water for injection was purchased from Fresenius Kabi, 0.9% NaCl from BBraun. 

[^18^F]F^−^ was produced as described in Section 4.1.2.

#### 4.2.3. Process Description

The target water containing [^18^F]F^−^ was trapped on the QMA-cartridge by passing the irradiated [^18^O]H_2_O through the QMA-cartridge into the vessel for [^18^O]H_2_O recovery in vacuo. Subsequently, [^18^F]F^−^ was eluted with 750 µL 0.075 M aqueous TBAHCO_3_-solution (Vial 1) into the reaction vessel using a vacuum. Drying was accomplished by azeotropic distillation using 1 mL of acetonitrile from Vial 2. After completing the drying process, 1.6 mg of Precursor 3 in 2 mL of DMSO from Vial 3 were added to the reaction vessel and heated for 10 min at 85 °C. The reaction mixture was then taken up in 10 mL 5% EtOH solution from Vial 4 and passed through the PS-H^+^ and C18_ec_ cartridges into the waste. Both cartridges were first washed with 23 mL of 5% EtOH solution from Vial 5 and then with 3 mL 30% EtOH solution from Vial 6 into waste to remove chemical and radiochemical impurities. The product was subsequently eluted with 4 mL 30% EtOH solution from Vial 7 into the pre-loaded collection vial (11 mL 0.9% saline with 100 mg sodium ascorbate as the stabiliser) and mixed with a stirrer. The final product solution of 15 mL was then transferred into a Class A isolator and sterile-filtered using a Millex-Cathivex GV 0.22-µm filter. 

### 4.3. Production of [^18^F]PSMA-1007 via Direct Substitution on GE TRACERlab MX and NEPTIS Mosaic-RS (SPE Purified)

#### 4.3.1. Setup of the Radiosynthesiser

**Figure 8:**
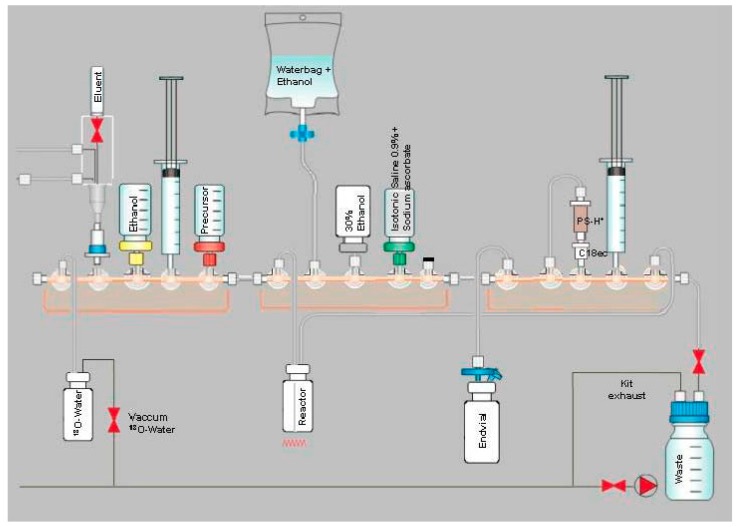
General setup of the radiosynthesiser for one-step production of [^18^F]PSMA-1007 with SPE cartridge purification on GE TRACERlab MX and the NEPTIS mosaic-RS system.

Both radiosynthesisers employ the same disposable 5-stop-cock manifolds equipped with syringes, silicone tubings and a glass reaction vessel. The general setup is depicted in Figure 8. For cartridge separations, a pre-conditioned Sep-Pak^®^ Light Waters Accell^TM^ Plus QMA cartridge, a Chromafix PS-H^+^ (L) cartridge (Macherey-Nagel, not pre-conditioned) and a Chromafix C18_ec_ (M) (Macherey-Nagel, not pre-conditioned) were installed on each radiosynthesiser (QMA on Position 2, PS-H^+^ and C18_ec_ on Position 13). On Position 3, a vial with 8 mL ethanol is placed. On Position 5, a vial containing a solution of 1.6 mg Precursor 3 in 2 mL DMSO is inserted. A 100-mL water bag containing 5.5 mL ethanol is connected to Position 7. For elution of the cartridges, a vial containing 8.5 mL of 30% aqueous ethanol is mounted on Position 8. On Position 9, a vial with 15 mL of 0.9% sodium chloride containing 100 mg sodium ascorbate is placed. Position 10 is not used and closed with a stopper. Two 30-mL syringes are attached on Positions 4 and 14. The inlet of the glass reaction vessel is connected to Position 6 and the outlet to Position 15.

#### 4.3.2. Reagents and Radionuclide Production

Precursor 3 and TBAHCO_3_-solution were prepared in-house at ABX GmbH (Radeberg, Germany). Acetonitrile (for DNA synthesis) and ethanol were acquired from Merck (Darmstadt, Germany). Anhydrous DMSO and sodium ascorbate were received from Aldrich. The 0.9% sodium chloride solution was obtained from BBraun. [^18^F]F^−^ was produced by irradiation of [^18^O]H_2_O (CIL) with 9.6 MeV proton beams by the ^18^O(p,n)^18^F nuclear reaction. Irradiations were performed with the GE Minitrace 700S cyclotron at ABX GmbH. 

#### 4.3.3. Process Description

The target water containing [^18^F]F^−^ was trapped on the QMA-cartridge by passing the irradiated [^18^O]H_2_O through the QMA-cartridge into the vessel for [^18^O]H_2_O recovery. Subsequently, [^18^F]F^−^ was eluted with 750 µL 0.075 M aqueous TBAHCO_3_-solution into the reaction vessel using a vacuum. Drying was accomplished by applying a flow of nitrogen on the inlet and vacuum on the reaction vessel outlet at elevated temperature. After completion of the drying process, 1.6 mg of Precursor 3 in 2 mL of DMSO from Position 5 were added to the reaction vessel and heated for 10 min at 5 °C. The reaction mixture was diluted with 4 mL 5.5% EtOH solution and passed through the PS-H^+^ and C18_ec_ cartridges into the waste. Both cartridges were first washed with 3 × 10 mL 5.5% EtOH solution and then with 3 mL 30% EtOH solution into waste to remove chemical and radiochemical impurities. The product was finally eluted with 5 mL 30% EtOH solution into the product vial by passing through a sterile Millex-Cathivex GV 0.22-µm filter and diluted with 15 mL 0.9% saline containing 100 mg sodium ascorbate, which was also passed through the sterile Millex-Cathivex GV 0.22-µm filter into the final product vial.

### 4.4. Production of [^18^F]PSMA-1007 via Direct Substitution on IBA SYTHERA+

#### 4.4.1. Setup of the Radiosynthesiser

**Figure 9:**
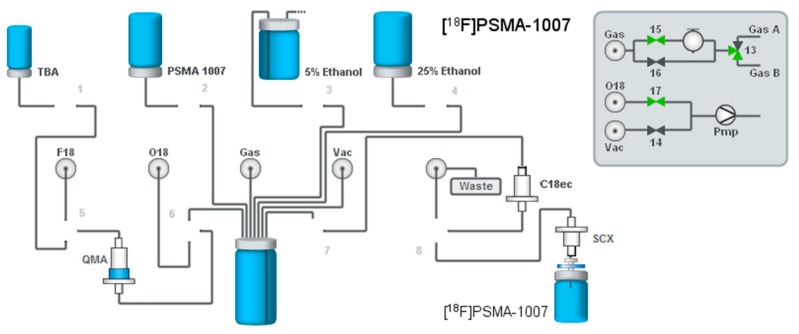
General setup of the radiosynthesiser for the one-step production of [^18^F]PSMA-1007 with SPE cartridge purification on the IBA SYNTHERA+ system.

A standard FDG IFP^TM^ was used for the radiosynthesis. The general setup is depicted in Figure 9. For cartridge separations, a pre-conditioned Sep-Pak^®^ Light Waters Accell^TM^ Plus QMA cartridge and a Macherey-Nagel C18ec (pre-conditioned with 2 mL ethanol and 10 mL water for injection) were installed on the radiosynthesiser. For final purification, a Sep-Pak^®^ SCX (Waters, pre-conditioned with 5 mL water for injection) was installed on top of the sterile filter of the final product vial. On the left front position, a vial with 600 µL TBAHCO_3_-solution was mounted. Twenty five percent ethanol for elution was mounted on the front right position (6.0 mL). The precursor vial containing 1.0 mg Precursor 3 dissolved in 1.5 mL DMSO was mounted on the rear left position. Furthermore, a vial with 33 mL 5% ethanol solution was placed in front of the synthesiser. The vial was vented with a 0.22-µm vent filter. Prior to the synthesis, 10 mL of 0.9% saline containing 75 mg sodium ascorbate were transferred into the sterile vial by passing through a Cathivex GV 0.22-µm filter.

#### 4.4.2. Reagents and Radionuclide Production

See Section 4.3.2.

#### 4.4.3. Process Description

The target water containing [^18^F]F^−^ was trapped on the QMA-cartridge by passing the irradiated [^18^O]H_2_O through the QMA-cartridge into the reaction vessel for [^18^O]H_2_O recovery. Subsequently, [^18^F]F^−^ was eluted with 600 µL 0.075 M aqueous TBAHCO_3_-solution into the reaction vessel using a vacuum. Drying was accomplished by applying a flow of nitrogen on the inlet and vacuum on the reaction vessel outlet at elevated temperature. After completion of the drying process, 1.0 mg of Precursor 3 in 1.5 mL of DMSO was added to the reaction vessel and heated for 10 min at 85 °C. The reaction mixture was then taken up in 4 mL 5% EtOH solution and passed through the C18ec cartridge into the waste. The cartridge was washed with 4 × 5 mL 5% EtOH and 1 × 3 mL 25% ethanol, which was transferred into waste. The product was then eluted with 5 mL 25% ethanol into the final vial already containing sodium chloride/sodium ascorbate by passing through a SCX cartridge and a sterile Millex-Cathivex GV 0.22-µm filter in series.

### 4.5. Quality Control

#### 4.5.1. Standard Procedures

The injection solution was tested for residual solvents and DIPEA by gas chromatography using a 6850 Series II Network GC System with GC Chem Station software Version 10.02 (Agilent Technologies, Waldbronn, Germany). The pH-value of the formulation was determined by potentiometry using pH/Ion Meter 692 (Metrohm AG). The test for endotoxins was conducted by the limulus amebocyte lysate test (LAL test) on an Endosave^®^ PTS (Charles River Laboratories International, Inc., Tokyo, Japan) and filter integrity using the bubble point test. Finally, a sample of the product formulation was tested for sterility post release by an independent institution using direct inoculation according to the current European Pharmacopoeia monographs.

#### 4.5.2. HPLC Analysis

Calibration of the HPLC systems was conducted by injection of non-radioactive PSMA-1007 reference standard using the respective gradients. The reference standard was provided by ABX. Identification and purity were checked by ^1^H-NMR and HPLC. Identification of [^18^F]PSMA-1007 was conducted by co-elution with the reference standard.

At DKFZ, HPLC analysis was performed on an Ultimate 3000 system with a variable wavelength detector RS 3000 (both Thermo Fisher Scientific, Schwerte, Germany) and a Gabi detector (Raytest, Straubenhardt, Germany) for radioactivity detection, equipped with a Chromolith performance C18ec 100 × 4.6-mm column (Merck). The system was controlled by Chromeleon software Version 7.1.2 (Dionex). For the analysis, a multi-step gradient was applied using acetonitrile (Solvent A) and 0.1% TFA (Solvent B): 5% A to 15% A in 1 min, then to 35% A in a further 9 min, then to 95% A in a further 2.5 min and finally back to 5% A in a further 2.5 min (A + B = 100%, flowrate = 3 mL/min, total run time = 15 min).

At ABX, HPLC analysis was performed on a Dionex Ultimate 3000 or Agilent 1200 system both equipped with a gamma-detector HERM LB500 (Berthold) for radioactivity detection and a Waters X-Terra C18 250 × 4.6-mm column. The systems were controlled by Chromeleon software Version 6.8. or 7.1.2. A multi-step gradient was applied using acetonitrile (Solvent A) and 0.1% TFA (Solvent B): 0–0.3 min, 20% A; 0.3–2 min to 30% A; then 30% A for 15 min, then to 95% A in 6 min; then 95% A for 2 min; back to 20% A in 1 min; and finally, 9 min at 20% A (A + B = 100%, flowrate = 1 mL/min, total run time = 35 min).

#### 4.5.3. Thin Layer Chromatography 

TLC was performed using TLC Silica Gel 60 F_254_ Alu sheets (5 × 7.5 cm). Approximately 1 µL of the [^18^F]PSMA-1007 injection solution was spotted at 1.5 cm, and the run length was 5 cm. Solvent for the development of the TLC plates was acetonitrile/water 60:40 *v*/*v*. The developed plate was analysed using a mini Gita detector system with Gina Star software Version 4.07 (both Raytest). 

For the residual tetrabutylammonium ion (TBA), the TLC spot test from Kuntzsch et al. [29] was used with slight modifications. TLC was performed using Polygram SIL G/ UV_254_ (Macherey-Nagel) 4 × 8 cm. The TLC chamber was conditioned with a 9:1-mixture of methanol and 25% ammonia (*v*/*v*). For preparation of the reference solution, 330 mg tetrabutylammonium hydroxide 30-hydrate (Mw: 799.93 g/mol; 386 mmol) were dissolved in 10 mL of a 9:1-mixture of water and ethanol (*v*/*v*) in a volumetric flask (equal to 10 mg TBA/mL; Mw (TBA): 242.5 g/mol). One hundred microlitres of the resulting mixture were diluted to 10 mL in a volumetric flask using a 9:1-mixture of water and ethanol (*v*/*v*) (reference solution; equal to 0.1 mg TBA/mL). TLC analysis was performed by applying two separate spots of 2 µL reference and test solution ([^18^F]PSMA-1007 formulation) each 10 mm from the bottom. The TLC plate was properly dried with a stream of air at ambient temperature and then developed in the TLC chamber up to 10 mm below the upper edge. After proper drying with a stream of air at ambient temperature, the plate was placed in a second chamber filled with a few crystals of iodine for 1 min. The reference standard should show a dark brownish spot approximately at R_f_ 0.2–0.3. If no spot appears, the iodine chamber can be gently heated from outside using a heat gun at 50–80 °C, which results in the formation of iodine vapors.

## 5. Conclusions

We have developed a new precursor including procedures for the GMP-compliant one-step radiosynthesis of [^18^F]PSMA-1007 injection solution on several commonly-used radiosynthesisers, replacing our low yielding two-step method. Using the new unprotected precursor, radiofluorination was achieved in excellent radiochemical yields of up to 80% after purification by simple cartridge extraction and short times (35–55 min) providing a radiosynthesis process technically comparable to that for the well-known [^18^F]FDG radiosynthesis. Upscaling to 50 GBq product per batch was successfully accomplished. The product batches meet the quality criteria according to the European Pharmacopoeia. This unique one-step radiofluorination approach was easily adapted for several radiosynthesisers to concomitantly pave the way for the clinical availability of [^18^F]PSMA-1007.

## Figures and Tables

**Table 1:** Recommended tests for the quality control (QC) of [^18^F]PSMA-1007 injection solution. LAL: limulus amebocyte lysate.

Parameter	Method	Acceptance Criteria
Appearance	Visual	Clear and colourless
Identity	HPLC	R_t_ ± 0.5 min of reference standard
Radiochemical purity	HPLC	≥95%
	TLC	≥95%
Radionuclidic purity	Half life	110 ± 5 min
	Gamma spectroscopy	511 keV ≥ 99.9% (post-release)
Chemical purity ^#^	HPLC	PSMA-1007: ≤0.1 mg/V_max_
		Any other impurity *: ≤0.1 mg/V_max_
		Sum of all impurities *: ≤0.5 mg/V_max_
	TLC	TBA: ≤2.6 mg/V_max_
	GC	Acetonitrile: ≤4.1 mg/V_max_
		DMSO: ≤50 mg/V_max_
		EtOH: ≤10% V/V
		Acetone: ≤50 mg/V_max_
pH	Potentiometric or strip indicator	4.5–7.5
Endotoxins	LAL test	≤175 IU/V_max_
Filter integrity	Bubble point test	≥3.5 bar (Cathivex-GV 0.22 µm)
Sterility	Post-release	Sterile (post-release)

* Assuming that the UV absorption is equivalent to PSMA-1007; ^#^ V_max_ was defined as a maximum of 10 mL per patient.
